# Enhancement Mechanism of Pt/Pd-Based Catalysts for Oxygen Reduction Reaction

**DOI:** 10.3390/nano13071275

**Published:** 2023-04-04

**Authors:** Xinqun Zhang, Jiaqi Wang, Yang Zhao

**Affiliations:** Key Laboratory of Cluster Science, Ministry of Education, Beijing Key Laboratory of Photoelectronic/Electrophotonic Conversion Materials, School of Chemistry and Chemical Engineering, Beijing Institute of Technology, Beijing 100081, China

**Keywords:** ORR, Pt/Pd-based catalysts, enhancement mechanism

## Abstract

The oxygen reduction reaction (ORR) is one of the key catalytic reactions for hydrogen fuel cells, biofuel cells and metal–air cells. However, due to the complex four-electron catalytic process, the kinetics of the oxygen reduction reaction are sluggish. Platinum group metal (PGM) catalysts represented by platinum and palladium are considered to be the most active ORR catalysts. However, the price and reserves of Pt/Pd are major concerns and issues for their commercial application. Improving the catalytic performance of PGM catalysts can effectively reduce their loading and material cost in a catalytic system, and they will be more economical and practical. In this review, we introduce the kinetics and mechanisms of Pt/Pd-based catalysts for the ORR, summarize the main factors affecting the catalytic performance of PGMs, and discuss the recent progress of Pt/Pd-based catalysts. In addition, the remaining challenges and future prospects in the design and improvement of Pt/Pd-based catalysts of the ORR are also discussed.

## 1. Introduction

During social development, the massive use of energy sources based on fossil fuels has led to serious environmental crises such as global warming and environmental pollution [1,2,3,4]. Fuel cells represented by proton-exchange membrane fuel cells have attracted extensive attention due to their high energy density, splendid energy conversion efficiency and pollution-free properties, and are expected to supply a new generation of sustainable clean energy for human development [1,5,6]. In addition, metal–air cells present a lower cost and higher energy density than lithium-ion batteries, and the working environment of the water system also promises higher safety [7,8]. The oxygen reduction reaction (ORR) is one of the key steps for electrochemical conversion processes, in which the ORR electrocatalyst has a crucial impact on energy conversion efficiency and performance [9,10]. Therefore, the development of ORR catalysts with a higher catalytic activity and more excellent durability is of great significance to promote the application of these sustainable energy systems.

In recent years, with the efforts of many research groups, a number of methods for synthesizing ORR catalysts have been developed. In particular, the development of density functional theory (DFT) technology has effectively found the heart that determines catalytic activity and has screened out a variety of ORR catalysts with great catalytic potential, such as non-noble-metal-based catalysts and noble metal catalysts (such as Pt/Pd-based catalysts) [6,11]. After several years of development, non-precious metal catalysts represented by M-N-C (M = Fe, Co) have made great progress and have even demonstrated higher catalytic performances than commercial platinum–carbon [9,12,13,14]. Additionally, compared with PGMs, non-precious metals have lower costs and broader sources. Nevertheless, PGMs cannot be replaced by other catalysts due to their impressive catalytic activity, even though there are some major issues such as scarcity and high cost. Meanwhile, the cost and loading of Pt/Pd can be effectively reduced by improving the catalytic performance [15,16,17,18,19,20]. Therefore, PGM catalysts are still attracting attention even though non-noble metal catalysts have made great progress.

**Figure 1:**
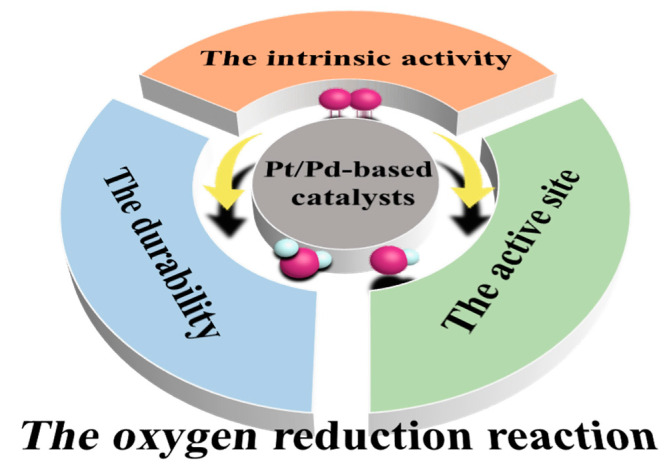
The Schematic of the optimizing strategies for Pt/Pd-based catalysts.

At present, the research focus regarding PGMs is to synthesize a catalyst with better performance in order to reduce the load of PGMs and their cost. Several reviews have been reported to summarize catalyst synthesis methods and structure–activity relationships. Considering that significant progress towards Pt/Pd-based ORR catalysts has been achieved, but with no systematic summary of the performance enhancement mechanism, this review focuses on the performance improvement strategy and mechanism of Pt/Pd-based catalysis. Firstly, the intermediate products and reaction processes in the ORR process are introduced, the factors affecting the catalytic kinetics and mechanisms are discussed, and the main ideas to improve the catalytic performance of the catalyst are summarized as increasing the intrinsic activity, the number of active sites and the durability. (Figure 1) Then, combined with the above theory, we focus on the recent progress in the synthesis of Pt/Pd-based catalysts and analyze the reasons for their excellent performance. Finally, we discuss the current barriers and challenges of Pt/Pd-based catalysts and give our own suggestions for overcoming these barriers.

## 2. The Kinetics, Mechanisms and Performance Parameters of Pt/Pd-Based Catalysts for ORR

It is believed that the ORR process can be mainly divided into two reaction pathways, one is the process of partial reduction to H_2_O_2_ or HO_2_^−^ with two-electron pathways [21,22], which would adversely affect the catalyst due to the low potential and strong oxidation ability. The other is the process of the complete reduction of oxygen to H_2_O or OH^−^ with four-electron pathways [2,23], which is regarded as the preferred reaction process. Therefore, this review focuses on the four-electron pathway. Due to the different reaction media and mechanisms, the four-electron reaction process of the ORR has the following two reaction pathways in acidic/basic solvents (step 1 and 2) [2,16,23]. Depending on the proton concentration of acidic/basic solvents, the essential difference is whether the hydrogen protons of protonation for O_2_ come from H_2_O or hydrogen ions in the solvent. According to the timing of O-O bond breaking, the reaction mechanism can also be divided into associative (Equations (1a) and (2a)) and dissociative (Equations (1b) and (2b)). Additionally, it is easy to conclude that the formation and decomposition of intermediates (M-OOH, M-O, M-OH) is the key to the reaction. The binding ability of the intermediate in the active site of the catalyst should not be too strong or too weak [6,16]. More concretely, if the interaction between the intermediates and the active site is too strong, then the decomposition of the intermediates and the reaction will be hindered, while a relatively weak interaction will limit the adsorption of oxygen and the formation of the intermediates. Therefore, the ideal catalysts should exhibit the appropriate interaction strength with the intermediates to ensure that the formation and dissociation of the intermediates can be carried out smoothly.

Reaction step 1 in acidic solvents:

(1a) $$O_{2}+\;M*\overset{H^{+}+e^{-}}{\to }M-\mathrm{OOH}\;\overset{H^{+}+e^{-}-H_{2}O}{\to }M-O\;\overset{H^{+}+e^{-}}{\to }M-\mathrm{OH}\;\overset{H^{+}+e^{-}-H_{2}O}{\to }M*$$

(1b) $$O_{2}+2M*\overset{}{\to }2M-O\;\overset{2H^{+}+2e^{-}}{\to }2M-\mathrm{OH}\;\overset{2H^{+}+2e^{-}-2H_{2}O}{\to }2M*$$

Reaction step 2 in basic solvents:

(2a) $$O_{2}+\;M*\overset{H_{2}O+e^{-}-{\mathrm{OH}}^{-}}{\to }M-\mathrm{OOH}\;\overset{H_{2}O+e^{-}-{\mathrm{OH}}^{-}}{\to }M-O\;\overset{H_{2}O+e^{-}-{\mathrm{OH}}^{-}}{\to }M-\mathrm{OH}\;\overset{-{\mathrm{OH}}^{-}}{\to }M*$$

(2b) $$O_{2}+2M*\overset{}{\to }2M-O\;\overset{2H_{2}O+2e^{-}-2{\mathrm{OH}}^{-}}{\to }2M-\mathrm{OH}\;\overset{-2{\mathrm{OH}}^{-}}{\to }M*$$

where M∗ is the catalytic active site.

DFT calculations are typically utilized to explore the free energies of the intermediates, active sites and catalytic mechanisms, and to provide a theoretical basis for the high ORR activity of catalysts. By calculating the oxygen adsorption energy (ΔE_O_) and catalytic activity of different materials, Nørskov and co-workers constructed a curve of the relationship between activity and ΔE_O_, which is known as the ‘‘volcano curve’’ of the ORR [6,16]. According to the curve, Pt and Pd are the closest to the vertex of the volcano, but not above it, indicating that the catalytic activity of Pt/Pd can theoretically be further enhanced. It is generally believed that ΔE_O_ is determined by the d-band center position (calculated with respect to the Fermi level) of Pt/Pd-based ORR catalysts [24,25]. Modulating the position of the d-band center plays a decisive role in improving catalytic properties, and the shift of the d-band center can be achieved by the ligand effect and strain effect [26,27,28]; the former can effectively change the density of states near the Fermi level, while the latter can adjust the energy of the d-band center. The combination of experiment and theory is essential to explain the formation and dissociation of intermediates and the process of the reaction, and to determine the active site and the rate-determining step of the reaction, which is important to optimize the catalyst activity and develop a high-efficiency catalyst.

## 3. Enhancement Strategies of Pt/Pd-Based ORR Catalysts

After years of development, Pt/Pd-based catalysts are still irreplaceable due to their unparalleled catalytic activity, even though the catalytic performance of non-noble metal catalysts has been significantly improved. The scarcity and high cost of Pt/Pd-based catalysts can be alleviated by improving catalytic performance and reducing catalyst loading [17,24]. There are three main strategies to improve the catalytic properties of Pt/Pd-based catalysts:

(i) Enhancing the intrinsic activity of Pt/Pd-based catalysts: The adsorption energies of the reactants and activation barriers are determined by the position of the d-band center, while the shift of the d-band center can construct the optimum model catalyst.

(ii) Increasing the exposed active sites of Pt/Pd-based catalysts: Theory and research of surface compounds indicate that a small number of unsaturated coordination atoms or defects on the surface of catalysts are catalytic active centers, while the bulk-like catalyst only acts as a carrier. Reducing the particle size and constructing well-defined microstructures are utilized to increase the active sites and enhance the efficiency of Pt/Pd-based catalysts.

(iii) Improving the stability and durability of Pt/Pd-based catalysts: Enhancing the durability of catalysts and increasing the service life of catalysts are also of positive significance to reduce the cost of catalysts.

According to above points, great efforts for highly efficient Pt/Pd-based catalysts have been achieved. Based on this, we will summarize the development and process of the properties for Pt/Pd-based catalysts.

### 3.1. Enhancing the Intrinsic Activity of Pt/Pd-Based Catalysts

The introduction of other transition metals to Pt/Pd-based alloys or core–shell structures (M_core_@Pt/Pd_shell_) can boost the ORR activity via optimizing the d-band center position [19,24,29,30,31,32,33]. On the one hand, heteroatoms can produce ligand effects with surrounding Pt/Pd atoms and adjust the electronic structure of catalytic active atoms through strong electronic coupling effects, which will undoubtedly affect the adsorption energy of Pt/Pd-based catalysts [26]. On the other hand, owing to mismatched lattice constants and different atomic radii between the heteroatoms and Pt/Pd, the resulting strain effects can also cause movements of the d-band center [6]. Studies have indicated that compression strains can reduce the adsorption energy between the intermediates and the active sites of catalysts, in which the dissociative process tends to occur due to the narrower atomic spacing, whereas tensile strains exhibit the opposite effect [34,35]. It is generally believed that compression strains have a positive effect on the ORR of Pt/Pd-based catalysts.

**Figure 2:**
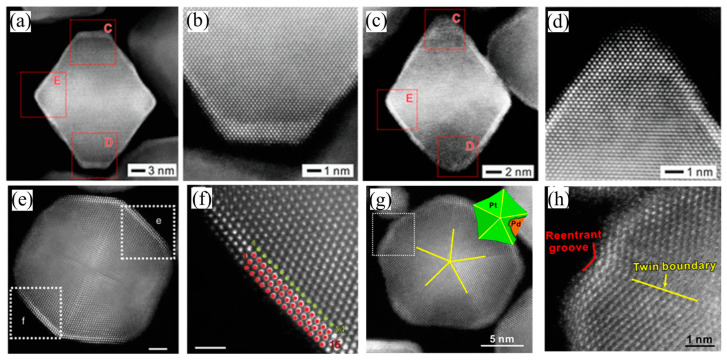
Structural analyses of the Pt/Pd bimetallic nanocrystals. (**a**–**d**) HAADF-STEM images of the Pd@Pt_2−3L_ octahedra synthesized using the polyol-based protocol and water, respectively. Reprinted with permission from Ref. [36]. Copyright 2015, ACS Nano. (**e**,**f**) Atomic-resolution HAADF-STEM images of Pd@Pt_2.7L_ icosahedra (green dots: Pd atoms; red dots: Pt atoms). Scale bar, 2 nm for (**e**) and 1nm for (**f**). Reprinted with permission from Ref. [37]. Copyright 2015, Nature Communications. (**g**) Atomic-resolution HAADF-STEM image and a model of the decahedron viewed along its 5-fold axis (inset). (**h**) Atomic-resolution AADF-STEM image taken from the corner marked by a box in (**g**), showing the presence of re-entrant groove at a vertex and twin boundary along a ridge. Reprinted with permission from Ref. [38]. Copyright 2015, Journal of the American Chemical Society.

Pt/Pd bimetallic nanocrystals are widely studied as ORR catalysts. With their similar face-centered cubic (fcc) structure and negligible mismatched lattice constants (with a mismatch of only 0.77%), Pd can easily form bimetallic nanocrystal alloys with single crystallinity, nanodendrites or core–shell structures with Pt [24]. Meanwhile, since platinum possesses promising catalytic ability and a higher cost than Pd, for decades it has usually been used as the main active metal for catalysis. To synthesize Pt/Pd nanodendrites or core–shell structures, seed-mediated growth may be the most powerful and productive strategy. In this regard, Xia and co-workers successfully synthesized Pd@Pt_nL_ (n = 2−5) core–shell octahedra using polyols and water by seed-mediated growth and explored the synthesis mechanism (Figure 2a–d) [36]. This work proved that the adsorption of OH on the surfaces of the octahedron could be optimized, promoting the hydrogenation of OH. Even though the lattice mismatch of Pd and Pt is negligible, the strains still exist and affect the catalytic process. In the same year, Xia’s group reported a Pd@Pt_2.7_ core–shell icosahedra with compressive strain by using palladium twins as the seed (Figure 2e,f) in a similar synthesis strategy [37]. Under the lateral constraints of the twin boundary and vertical relaxation, the platinum layers could only relax towards the twin plane along the vertical relaxation direction and form a corrugated structure with compressive strain. DFT calculations suggested that the binding of the catalyst and OH was weakened under the action of ligand and strain, which showed a positive effect on the catalytic process. In the same year, they fabricated multiply twinned Pd@Pt concave decahedra using palladium twins as a crystal seed by controlling the synthesis process (Figure 2g,h) [38]. At higher reaction temperatures, the Pt atoms were preferentially deposited onto the (100) and (211) facets on the edges/ridges of a decahedron and then diffused across the surface along the edge/ridge surface to form a concave structure. The unique concave structure induced the decahedrons to present high-index facets, which caused numerous high densities of steps, edges, and low-coordinated surface atoms. In addition, combined with the ligand effect and strain effect arising from the lattice mismatch, the catalyst showed excellent catalytic performance.

**Figure 3:**
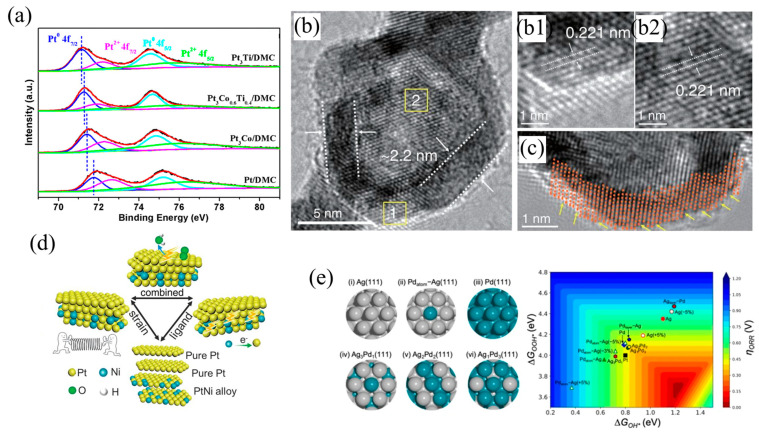
(**a**) Pt 4f XPS spectra of Pt_3_Co_1−x_Ti_x_ and Pt/DMC. Reprinted with permission from Ref. [39]. Copyright 2022, ACS Catalysis. (**b**) Enlarged TEM image and (**b1**,**b2**) the corresponding HRTEM images of the areas marked by yellow squares. (**c**) Atomic-resolution HRTEM image in the edge of Pt–Ni nanocages. (**d**) Illustration of the synergistic effects derived from both the lattice strain and ligand effects in the catalysts. Reprinted with permission from Ref. [19]. Copyright 2019, Nature. (**e**) Ag-Pd active site models investigated by DFT for their effect on ORR activity and oxygen reduction reaction (ORR) activity map showing overpotentials. Reprinted with permission from Ref. [30]. Copyright 2021, Nature Communications.

From the perspective of economic development, the development and usage of noble metals (e.g., Pt, Pd) is mainly affected by their costs. In recent decades, the cost of platinum has begun to decrease gradually, becoming even lower than palladium. Therefore, the use of transition metals (such as Fe, Co, Ni, Cu, Pb, Ag) instead of Pd may not only reduce the cost of the catalyst, but also help to improve the catalytic performance of Pt/Pd-based catalysts. A variety of transition metal element options opens various possibilities for the synthesis of catalysts and the ligand effect. Most importantly, these elements have more obvious atomic radius differences and mismatched lattice constants with Pt/Pd, which may lead to more severe structural strains affecting the catalytic process. Zhao and co-workers demonstrated an efficient ternary catalyst consisting of Pt_3_Co_1−x_Ti_x_ supported on mesoporous carbon derived from ZIF-8 by an improved impregnation reduction method [39]. The electron regulation effect of Ti on Pt was investigated by changing the substitution ratio of Ti to Co. Experimental results and DFT calculations demonstrated that the partial substitution of Ti for Co could adjust the electronic structure of Pt, optimize the adsorption energy of the intermediates on the surface of catalysts, and thus improve the catalytic performance. The improvement of performance was mainly attributed to the adjustment of the ligand effect of Ti on the system (Figure 3a). Co-chemical reduction in oil-phase synthesis at an elevated temperature is the most common and feasible method to synthesize Pt/Pd–M (M = transition metal elements, Fe, Co, Ni, Cu, Pb, Ag and so on) alloys. With this approach, Xia’s group prepared bunched 1D Pt–Ni alloy nanospheres in the oil phase, which could be converted into nanocages composed of a Pt skin and a residual Pt–Ni alloy below this skin by further acid etching [19]. The structural defects caused by acid etching were the source of crystal strain (Figure 3b,c). In addition, the ratio of nickel to platinum also affected the catalytic performance, suggesting the existence of a coordination effect. DFT calculations revealed that the synergistic effects of the strain and coordination environment led to the superior ORR activity, and the appropriate Pt/Ni ratio provided a properly weakened Pt–O binding strength. The catalytic activity of palladium can also be improved by tuning the intrinsic strain and ligand effect (Figure 3d). Jaramillo and co-workers prepared Ag-Pd bimetallic thin films via electron-beam physical vapor co-deposition [30]. DFT calculations showed that ligand effects were consistent with the experimental results, whereas the strain effect had the opposite relationship. This phenomenon indicated that the ligand effect on the optimization of the activation energy was the main factor to improve the performance of the catalyst (Figure 3e).

**Figure 4:**
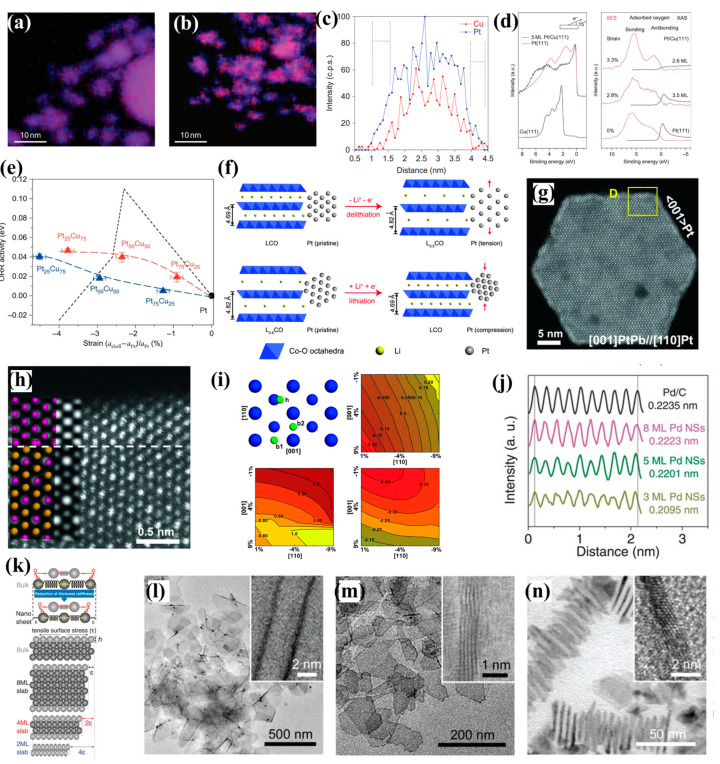
Elemental maps (**a**,**b**) and line profiles (**c**) of Pt−Cu bimetallic nanoparticle precursors and dealloyed active catalysts. (**d**) Surface science XAS and XES studies of single-crystal model systems that mimic dealloyed bimetallic core–shell structures. (**e**) Experimental and predicted relationships between electrocatalytic ORR activity and lattice strain. Reprinted with permission from Ref. [40]. Copyright 2010, Nature Chemistry. (**f**) Schematic of the lattice constant change in Li_x_CoO_2_ substrates and how the lattice strains are induced to Pt NPs. Reprinted with permission from Ref. [35]. Copyright 2016, Science. (**g**) HAADF-STEM image from out-of-plate view. (**h**) High-resolution HAADF image from the selected area in (**g**). (**i**) DFT calculations of oxygen adsorption energy. Reprinted with permission from Ref. [41]. Copyright 2016, Science. (**j**) The intensity profile and calculated average d-spacing of (111) planes. (**k**) Mechanism of the generation of intrinsic strain in 2D transition metal nanosheets. **h** is the height of an atomic layer. (**l**–**n**) TEM images of as-prepared Pd nanosheets (NSs) with average thicknesses of 3 ML (**k**), 5 ML (**l**), and 8 ML (**m**), with insets depicting typical structures. Reprinted with permission from Ref. [42]. Copyright 2019, Science.

The ligand and strain effects normally exist simultaneously in most alloy catalysts, where the strain effect has a greater effect because it has a long region of influence (up to 6 monolayers from the surface for the strain effect and about 1–3 monolayers for the ligand effect) [34]. Therefore, the influence of the ligand effect on catalysis could be avoided by constructing a core–shell structure with thick shell, so that the promoting effect of strain on catalysis could be explored and understood. Strasser and co-workers demonstrated dealloyed core–shell structures using liquid metal–salt precursor impregnation and acid etching [40]. The Pt–Cu bimetallic nanoparticle precursors were synthesized by freeze-drying, thermal annealing of copper precursors and commercial platinum electrocatalyst powders. Subsequently, the catalysts were further pretreated by cyclic voltammetry (CV) in 0.1 M HClO_4_ solution, during which a large amount of Cu was lost from the alloy nanoparticles, and these bimetallic nanoparticles were successfully converted into dealloyed nanoparticles with thick platinum-rich shell (Figure 4a–c). The lattice mismatch between the Pt shell and the Pt–Cu core led to a reduced Pt–Pt interatomic distance in the shell, indicating that the Pt shell was subject to compressive strain (Figure 4d). The regulation of the electronic structure was reduced owing to the shield of the thick shell layer, and the ligand effect could be ignored, so that the strain effect could be studied separately. Experiments and calculations showed that the antibonding state of oxygen 2p and platinum 5d changed from higher than the Fermi level to lower than the Fermi level, and the adsorbate bond weakened with the addition of compressive strain. The compression strain optimized the d-band structure of Pt atoms, and weakened the adsorption energy of reactive intermediates, which led to the increase in catalytic activity (Figure 4e). Cui’s group reported a method to control the lattice strain of Pt directly and continuously [35]. The volume of Pt nanoparticles supported on Li_x_CoO_2_ changed during the lithiation or delithiation process of Li_x_CoO_2_, resulting in the corresponding lattice strain (Figure 4f). With this method, a 5% adjustment of tensile or compressive strain was achieved. Both experimental results and DFT calculations showed that compressive strains weakened OH* binding and promoted the departure of the final product, which was favorable for the reaction, but tensile strains had an opposite and unfavorable effect on the catalytic process. Huang and co-workers presented a class of platinum–lead core @ platinum shell (PtPb@Pt)/nanoplate catalysts that exhibited large biaxial strains (Figure 4g,h) [41]. The SAED and HRTEM revealed that the edge–Pt and top (bottom)–Pt (110) facets exhibited large tensile strains (7.5%). The effect of the ligand on catalysis was ignored due to the thick shell. It is generally accepted that compressive strain is favorable for catalysis, but this catalyst with large tensile strains showed unusual catalytic properties. DFT confirmed that the low-coordinated atoms of platinum could be activated by larger tensile strains to optimize the adsorption strength of oxygen on the Pt (110) facet (Figure 4i).

Normally, the strain is induced by the heterogeneous matrix due to the mismatched lattice, which means the addition of heteroatoms is required, and the coordination effect can have an impact on the catalytic performance, even if this effect can be negligible. Wang and co-workers reported a strategy for synthesizing two-dimensional pure Pd nanosheets with compressive strain [42]. The ultra-thin nanosheets with 1–12 monolayers were successfully synthesized by controlling the reaction conditions. Due to the ultra-thin structure, the nanosheets were subject to surface stress, which resulted in compressive strain, and the strains were inversely proportional to the thickness (Figure 4j–n). DFT calculations elucidated that the compressive strain weakened the adsorption strength of oxygen and improved the catalytic performance, which was very similar to the case with Pt surfaces.

In addition, the synergistic effect for the various components of catalyst can also improve the catalytic performance. This effect may not improve the intrinsic catalytic activity of the catalyst, but it can have a positive impact on catalysis by improving the conductivity of the catalyst, optimizing the reaction process and the interface hydrophilicity, enhancing the mass transport, etc. [33,43,44,45,46,47]. On the one hand, the support material may produce strong electron coupling with the catalyst, improve the electronic structure of the catalyst and enhance the catalytic activity of the catalyst. On the other hand, the support material may have a certain optimization effect on the catalytic process and have a positive impact on the reaction kinetics [43,46].

### 3.2. Increasing Exposure of Catalytic Active Sites

Increasing the exposure of catalytic active sites can improve the utilization efficiency of precious metals, which is of great significance for reducing the cost and loading of Pt/Pd. It is necessary to maximize the number of catalytic active sites by surface engineering. The electrochemical active surface area (ECSA) is usually performed to measure the number of active sites. At present, the main strategies to increase the number of active sites are reducing the particle size, constructing special morphologies, exposing the active crystal face, and so on.

**Figure 5:**
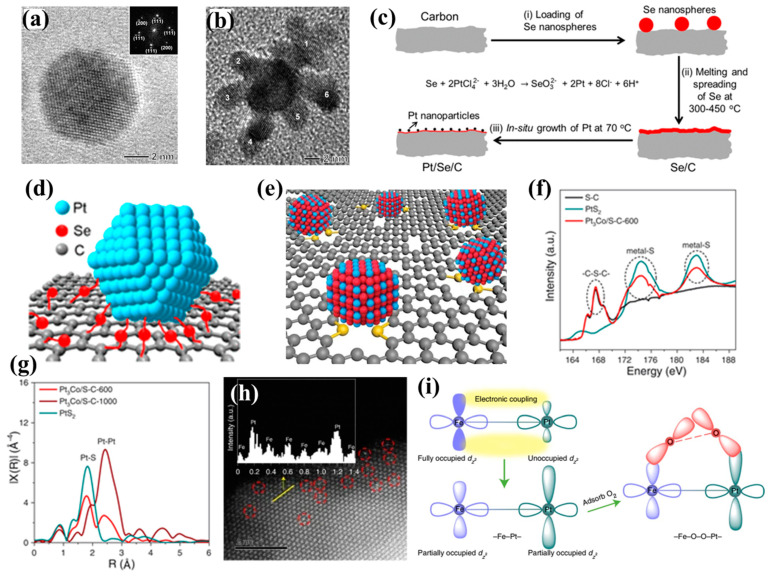
HRTEM image of Pd seed (**a**) and Pd−Pt nanodendrite (**b**). Reprinted with permission from Ref. [20]. Copyright 2009, Science. (**c**) Schematic illustration of a Pt−Se−C catalyst. (**d**) Schematic illustration showing how the Pt particle is anchored to the surface of a carbon support through the Pt−Se−C linkage. Reprinted with permission from Ref. [50]. Copyright 2019, Nano Letters. (**e**) Schematic illustration of the sulfur-anchoring synthetic approach. (**f**,**g**) XANES spectra and Fourier-transformed EXAFS data indicating the formation of Pt−S bonds. Reprinted with permission from Ref. [10]. Copyright 2021, Science. (**h**) An aberration-corrected HAADF–STEM image of Pt_1_–Fe/Fe_2_O_3_ (012), with single–site platinum marked by red dashed circles. (**i**) A schematic diagram of Pt–Fe electronic coupling effect for O_2_ activation. Reprinted with permission from Ref. [52]. Copyright 2021, Nature Energy.

Among them, reducing particle size is the most effective measure to increase the specific surface area of a catalyst and the exposure of active sites [4,17], which is also an important strategy to improve the performance of commercial catalysts. However, it is difficult to reduce the size of a catalyst to a small enough size in actual production (typically, below 5 nm) [48,49], because the small size of nanoparticles has a high reaction tendency, leading to agglomeration or sintering. Nanoparticles with small sizes can be synthesized more easily in the oil phase, which can provide abundant surfactants that limit the growth and aggregation of nanocrystals. Xia’s group successfully prepared Pd–Pt bimetallic nanodendrites by using ultra-fine Pd nanoparticles synthesized from the oil phase as the template (Figure 5a,b). Although the size of nanodendrites was larger than 10 nm, the average diameter of Pt branches was ~3 nm [20]. This special structure exhibited abundant crystal surfaces with high-index stepped Pt, which effectively improved the catalytic active area. However, the development of synthesis in the oil phase is limited by the cost of solvents and surfactants. The thermal reduction of a solid phase is an ideal synthesis method due to its simple operation process and low cost, but this strategy has long been plagued by aggregation and sintering. Xia’s group synthesized an ultra-fine platinum catalyst with a diameter of less than 2 nm using selenium (Se) as reducing agent and linker (Figure 5c) [50]. The initial ECSA of the catalyst was up to 231 m^2^ g^−1^_Pt_, more than three times that of the commercial catalyst (66.5 m^2^ g^−1^_Pt_). The linking effect of Se played an important role in improving the dispersion of Pt and alleviating the aggregation of particles (Figure 5d). Liang and co-workers used a similar idea to prepare a series of <5 nm diameter platinum alloy catalysts supported by sulfur-doped carbon, and this kind of catalyst had excellent resistance to sintering at high temperatures [10]. The high surface area and abundant S–C provided sufficient anchorage for platinum alloys (Figure 5e). The spectra of X-ray photoelectron spectroscopy (XPS) and X-ray absorption near edge structure (XANES) verified the presence of a large number of Pt–S bonds in these catalysts (Figure 5f,g), which enhanced the adhesion strength of the Pd-based alloy on the carbon supports, and interparticle sintering and agglomeration were inhibited.

The utilization efficiency of platinum atoms can be maximized by reducing Pt/Pd nanoparticles to single atoms [15,51]. Since single atoms cannot exist alone and need to be loaded onto supports, the strong electron coupling effect between supports and single atoms also affects the catalytic performance. Zou’s group constructed a single-site Pt–Fe pair by loading atomic Pt on α-Fe_2_O_3_ (012) facets, achieving superior ORR performance (Figure 5h) [52]. DFT calculations revealed that Pt–Fe pair sites could cooperatively adsorb O_2_ and dissociate the O=O bond, and the strong electron coupling effect promoted desorption of OH from the Pt site (Figure 5i).

The synthesis of nanomaterials with specific morphologies is also an effective strategy to enhance catalytic active sites. In past decades, noble metal nanomaterials with specific morphologies, such as nanowires, nanosheets, nanocages, nanoframes, and nanodendrites, are some of the most intensively studied nanomaterials due to their unique structures [49,53,54].

**Figure 6:**
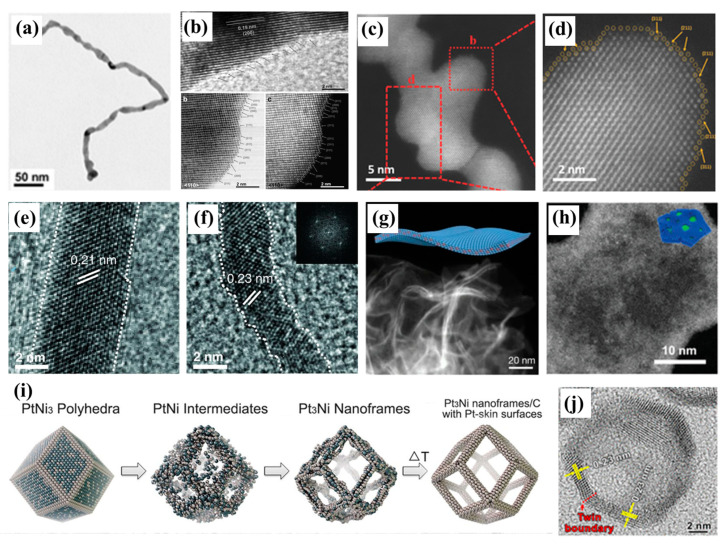
(**a**,**b**) TEM and HRTEM images of 1D Pt–Ni nanostructures. Reprinted with permission from Ref. [56]. Copyright 2015, Advanced Materials. (**c**,**d**) HAADF-STEM images of zigzag-like Pt–Fe nanowires. Reprinted with permission from Ref. [31]. Copyright 2018, Advanced Materials. HRTEM images of Pt–Ni alloy nanowires (**e**) and jagged Pt nanowires (**f**). Reprinted with permission from Ref. [11]. Copyright 2016, Science. HAADF-STEM images of Pd–Mo bimetallene (**g**) and Pd metallene with atomic-scale cavities (**h**). Reprinted with permission from Ref. [58]. Copyright 2019, Nature. Reprinted with permission from Ref. [59]. Copyright 2022, Advanced Materials. (**i**) Schematic illustrations of the samples obtained at four representative stages during the evolution process from polyhedral to nanoframes. Reprinted with permission from Ref. [61]. Copyright 2014, Science. (**j**) HRTEM image of Pt icosahedral nanocages. Reprinted with permission from Ref. [60]. Copyright 2016, Nano Letters.

The one-dimensional (1D) nanostructures represented by nanowires exhibit excellent properties such as inherent anisotropic morphology, high flexibility and high surface area [1,55]. In addition, crisscrossed metal nanowires can facilitate electron transport in the catalyst system. Pt/Pd-based nanowires are an ideal structure to improve the utilization efficiency of Pt/Pd. Huang’s group reported a multimetallic Pt-based 1D nanostructure with superior properties (Figure 6a) [56]. The surface of the nanowires presented a large number of low-coordination atoms due to the unusual one-dimensional structure, which was considered to be the catalytic active site, and had excellent catalytic activity (Figure 6b). Further optimizing the structure of the nanowires and synthesizing nanowires with an uneven surface could further improve the specific surface area and catalytic active area of the catalysts. Guo’s group presented zigzag-like Pt–Fe nanowires with high-index faceted Pt skin (Figure 6c) [31]. The special zigzag structure improved the exposure degree of the high-index facets, and thus improved the catalytic efficiency. The dealloying of multimetallic Pt-based nanowires can provide more abundant low-coordination atoms due to the departure of atoms attached to Pt (Figure 6d). Duan’s group prepared Pt/NiO core–shell nanowires in oil phase, and these nanowires could be converted into Pt–Ni alloy nanowires through a thermal annealing process in an argon/hydrogen mixture [11]. Then, the nanowires were transformed into jagged Pt nanowires via electrochemical dealloying (Figure 6e,f). Compared with the more relaxed surface, the undercoordinated rhombus-rich surface configurations of the jagged nanowires could provide abundant catalytic active sites for the catalytic process. The highly stressed low-coordination atoms on the surface effectively improved the catalytic activity.

Ultra-thin two-dimensional (2D) materials have also received extensive attention due to their unique structural properties [57]. Guo’s group presented curved ultra-thin Pd–Mo nanosheets named Pd–Mo bimetallene (Figure 6g) [58]. The Pd–Mo bimetallene catalysts had an ECSA of 138.7 m^2^ g^−1^_pd_ owing to their ultra-thin character. Defect engineering has been widely used in the design of catalysts for two-dimensional materials. On the premise of keeping the structure intact, etching can be used to create a large number of vacancy defects in the surface, which means more exposed active sites. Meanwhile, a large number of low-coordination atoms caused by defects generally have excellent catalytic performance. Guo’s group successfully synthesized Pd ultra-thin nanosheets with atomic-scale cavities [59]. Due to the presence of cavities, the nanosheets exhibited more exposed edge active sites (Figure 6h).

Different from 1D and 2D structures, three-dimensional (3D) structures (>5 nm) are considered to be unfavorable for the improvement of specific surface area and catalytic active area [49,60]. The aforementioned dilemma can be addressed by constructing nano-hollow or nanoframe structures, as in the strategy mentioned above for the Pt–Ni nanocages prepared by acid etching. Yang and co-workers synthesized Pt_3_Ni nanoframes converted from PtNi_3_ polyhedra by interior erosion (Figure 6i) [61]. The widths of the 24 edges that made up the frame were only 2 nm, suggesting that the framework had abundant reactive sites.

The order of catalytic activity for the Pt crystal face is Pt(100) << Pt(111) < Pt(110). Pt(111) and Pt(110) exhibit excellent catalytic activity [62,63,64,65]. It is also an effective way to increase the active area to expose the more active crystal surface by controlling the arrangement of atoms. Xia’s group reported the synthesis of Pt-based icosahedral nanocages whose surface was enclosed by both (111) facets and twin boundaries (Figure 6j) [60]. The nanocages exhibited excellent catalytic performance, which could be ascribed to the exposed (111) facets and twin boundaries.

### 3.3. Optimizing Durability of Pt/Pd-Based Catalysts

The durability of the catalyst is also an important index to measure the catalyst performance [5,66]. Catalysts are generally required to withstand more than 10,000 accelerated deterioration tests in most studies, and an excellent catalyst can withstand more than 30,000 cycles. Excellent durability can maximize the service life of the catalyst, thus reducing the operating cost of the whole life cycle of the device. The factors affecting catalyst durability can be summarized as follows: (1) detachment of catalyst from the system; (2) dissolution and aggregation of Pt nanoparticles caused by the Ostwald ripening process; (3) dealloying in acidic solvents.

**Figure 7:**
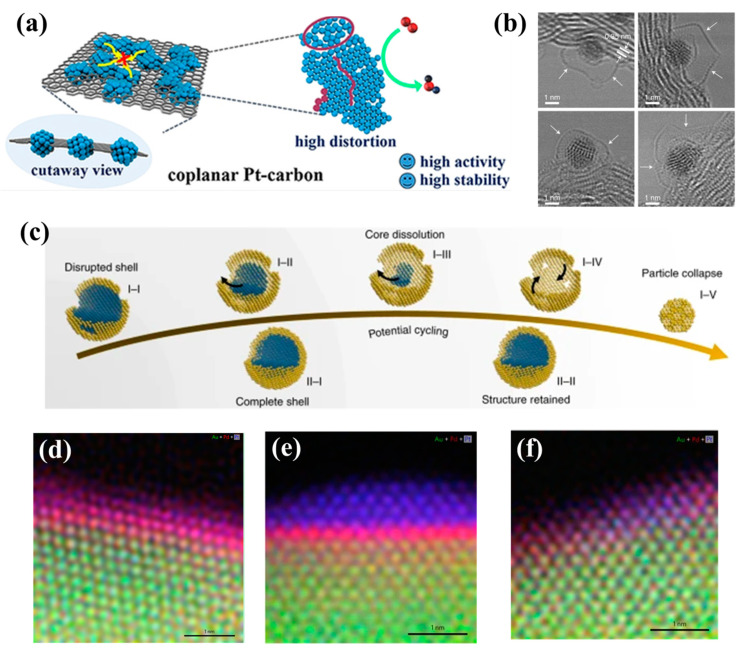
(**a**) Schematic illustration of two-dimensional coplanar Pt–carbon nanomeshes. Reprinted with permission from Ref. [67]. Copyright 2021, Angewandte Chemie International Edition. (**b**) Bright-field STEM images highlight the enclosure of ultra-fine nanoparticle by graphene nanopockets (indicated by white arrows). Reprinted with permission from Ref. [18]. Copyright 2022, Nature Nanotechnology. (**c**) Schematic showing the evolution of partially and fully coated core–shell particles during potential cycling. Reprinted with permission from Ref. [68]. Copyright 2020, Nature Materials. (**d**–**f**) Atomically resolved elemental mapping of the surfaces of fresh NPG–Pd–Pt, NPG–Pd–Pt_10,000_ and NPG–Pd–Pt_30,000_ electrocatalysts. Reprinted with permission from Ref. [69]. Copyright 2022, Nature Energy.

The optimization of the bonding between nanoparticles and the supports can effectively inhibit the detachment and the aggregation of particles, such as the interaction of Se or S–C with the nanoparticles mentioned above. Xun Hong and co-workers reported 2D coplanar Pt-carbon nanomeshes (NMs) composed of highly distorted Pt networks (Figure 7a) [67]. The unusual coplanar structure enhanced the interaction between carbon and Pt nanoparticles and restrained the tendency of Pt dissolution and aggregation. The encapsulation of a nanopocket is also an effective measure to protect nanoparticles. Huang’s group demonstrated the design of a graphene-nanopocket-encaged platinum cobalt (PtCo–Gnp) nanocatalyst (Figure 7b) [18]. The graphene nanopockets restricted catalyst coalescence and retarded oxidative dissolution, diffusion and the Ostwald ripening process.

The dissolution of Pt/Pd can be alleviated by introducing transition metal elements and constructing a polymetallic catalyst. Heteroatoms atoms can not only produce strong bonding with Pt/Pd atoms to alleviate the dissolution of Pt/Pd, but also increase the dissolution potential of Pt/Pd due to the difference in electrochemical activity. However, the corrosion and loss of heteroatoms (such as Cu, Ag, Co, or Ni) in an acidic environment can also lead to the weakening or disappearance of the ligand and strain effects, resulting in the decrease in catalytic performance.

The protection of the Pt shell in a core–shell (M_core_–Pt/Pd_shell_) structure is an effective measure to protect the core from dissolution. Marc Ledendecker and co-workers explored the protection effect of different thicknesses of Pt layers on the core of TiWC (Figure 7c) [68]. Benefiting from the protection of the thin layer and the stable interaction between the Pt shell and the TiWC core, the core–shell nanoparticles completely covering the Pt shell could maintain the core–shell structure and the atomic composition was stable after over 10,000 potential cycles. However, the position of each component atom in the core–shell structure is not fixed, and the internal atoms may diffuse to the surface during the catalytic process. Yi Ding and co-workers presented an unsupported nanoporous gold-based cathode electrocatalyst with a sub-nanometer Pd and Pt overlayer coating (NPG–Pd–Pt). This study confirmed that the original Pt–Pd bimetallic structure gradually evolved into a highly stable and active uniform Pt–Pd–Au trimetallic surface after the long cycle process (Figure 7d–f) [69]. The main reason for the degradation of the catalytic performance of core–shell catalysts was that the atoms in the core migrated to the surface and dissolved. To this end, supplementing the dissolved component of alloy or anchoring the heteroatoms firmly in the core could solve this problem [53]. Guo’s group achieved Au–Co–PtCoAu core–interlayer–shell multilayer structures. The interlayer Co could replenish the possible losses during the ORR process, which improved the durability of the catalyst. Huang and co-workers synthesized Mo-doped Pt-Ni/C octahedral nanoparticles [70]. The Mo oxides on the surface prevented the migration of Ni to the surface and protected the subsurface Ni from acid dissolution. Sasaki and co-workers reported a nitrogen-doped intermetallic Pt–Ni catalyst [71]. The Ni_4_−N formation and the unique intermetallic structure effectively prevented Ni dissolution from the core. Li and co-workers synthesized Pt_2_CuW_0.25_ ternary alloy nanoparticles; the stronger bonding between W and Pt/Cu atoms was the origin of the stability.

## 4. Discussion

Pt/Pd-based materials are one of most promising catalysts of the ORR and show broad application prospects. Over the past few decades, the catalytic mechanism has approached perfection as great research efforts have been undertaken. This review systematically summarizes recent research achievements in the improvement of catalysts, and classifies them into intrinsic activity, active site, and durability according to the promotion mechanism. Furthermore, combined with the reaction mechanism and kinetics, we discuss the factors affecting catalysis and strategies to improve catalytic performance.

In spite of these achievements, the application and development of Pt/Pd-based catalysts remain challenging:

First, cost is still the main challenge that limits the wide application of catalysts. The development of single-atom catalysts has provided a solution to this problem, but it is generally considered that the active surface (Pt/Pd (111) or (110)) is the catalytic active site. Zou’s work has provided guidance for improving the performance of platinum single-atom catalysts. The strong electronic coupling between the supports and the single atoms can be used to adjust the electronic structure of the single atoms and optimize the adsorption energy of the intermediates.

In addition, it is necessary to simplify and reduce the cost of the synthesis process. Some synthesis strategies for reactions in the oil phase require expensive solvents and surfactants, and tedious steps need to be performed to remove the surfactant and residual solvent. The solid-phase synthesis method is very suitable for large-scale industrial production due to its simplicity, rapidity and low cost, but it is still necessary to pay attention in order to avoid the sintering and agglomeration of nanoparticles in the synthesis process.

Last but not least, most of the research remains in the experimental stage. The rotating disk electrode (RDE) level performance cannot fully reflect its performance in practical applications. Although a large number of catalyst applications in devices have been reported and their performances have been tested, there are still many problems to be solved, which require the collaboration of multidisciplinary researchers.

## Data Availability

Data sharing not applicable. No new data were created or analyzed in this study. Data sharing is not applicable to this article.
